# Slow Release of GA_3_ Hormone from Polymer Coating Overcomes Seed Dormancy and Improves Germination

**DOI:** 10.3390/plants12244139

**Published:** 2023-12-12

**Authors:** Alexandra J. S. Larson, Maureen M. Cartwright, Whitney D. Jones, Katrina Luce, Mei-Yu Chen, Kate Petersen, Shannon V. Nelson, David J. Michaelis, Matthew D. Madsen

**Affiliations:** 1 Department of Plant and Wildlife Sciences, Brigham Young University, Provo, UT 84602, USA; ajsetelin@gmail.com (A.J.S.L.); whitdlarson@gmail.com (W.D.J.); katrina.luce.2@gmail.com (K.L.); danny48910@gmail.com (M.-Y.C.); shannon_nelson@byu.edu (S.V.N.); 2 Department of Chemistry and Biochemistry, Brigham Young University, Provo, UT 84602, USA; katempet@student.byu.edu (K.P.); dmichaelis@chem.byu.edu (D.J.M.)

**Keywords:** gibberellic acid, physiological dormancy, seed coating, penstemon, restoration

## Abstract

Seed dormancy often hinders direct seeding efforts that are attempting to restore degraded landscapes. Gibberellic acid (GA_3_) can be applied to physiologically dormant seeds to induce germination, but this hormone is rarely effective, as it can degrade or be leached from the seed. We tested different polymer matrixes (polylactic acid, polyvinylpyrrolidone, and ethylcellulose) to apply and slowly release GA_3_ to the seed. These polymers were tested as seed coatings in either a powder, liquid, or a combination of powder and liquid forms. We found that a liquid ethylcellulose/GA_3_ coating generally outperformed the other polymers and applications methods using our test species *Penstemon palmeri*. With this top-performing treatment, seed germination was 3.0- and 3.9-fold higher at 15 °C and 25 °C, respectively. We also evaluated the liquid ethylcellulose/GA_3_ coating on *P. comharrenus*, *P. strictus*, *P. pachyphyllus*, and *P. eatonii*. Again, the coating had a strong treatment response, with the degree of difference related to the relative level of dormancy of the species. Growth studies were also performed in pots to ensure that the side effects of GA_3_ overdosing were not present. Here, we found minimal differences in root length, shoot length, or biomass between plants grown from untreated and GA_3_-coated seeds.

## 1. Introduction

The need to restore damaged ecosystems has never been greater [1,2]. Native landscapes have been degraded worldwide, harming human populations and global economic output [1,3]. There is an urgent need for active restoration to reclaim these disturbed lands and maintain their ability to provide ecosystem services that will sustain human needs through the 21st century [1,4].

The restoration of native systems is commonly performed by revegetating landscapes through direct seeding efforts or planting nursery-grown seedlings. Direct seeding is the most common method for restoring native vegetation across large areas [5,6,7]. Yet, this technique commonly has low success rates, which leaves sites with low biodiversity, or it can result in complete seeding failure [8,9]. Seeding failure can be caused by various biotic and abiotic challenges [8,10]. Seed dormancy (and associated low germination) is one of the largest bottlenecks limiting the establishment of native species in restoration projects [3,10,11,12]. This seed attribute is a beneficial adaptation that allows for the long-term survival of the species within a community, but it can limit restoration success because seeds may take several seasons to lose dormancy [11]. If the seeded species do not occupy the site quickly during restoration, there may not be an opportunity in the future, particularly in areas with significant weed pressure or on sites with high soil erosion [5].

Physiological dormancy is the most common type of dormancy and is typically mediated, at least in part, through a chemical interplay between the growth hormone regulators abscisic acid (ABA) and gibberellins (GA) [13,14]. Abscisic acid induces dormancy, and GA plays a role in dormancy release and the promotion of germination [15]. Seed dormancy is maintained when there is a high ABA:GA ratio. Environmental factors, such as light, moisture, temperature, and seed age, can lower ABA levels, which results in an increase in GA biosynthesis and ABA catabolism. Dormancy is then released when there is a net shift to low ABA:GA ratios. Besides hormone content and synthesis, the shift from dormancy to the nondormant state in many seeds is marked by a reduction in ABA sensitivity and heightened responsiveness to GA.

For many species with physiological dormancy, it is often possible to preemptively break seed dormancy by adding GA to the seed, which increases the endogenous levels of GA relative to ABA [16,17]. The addition of GA to the seed may also promote the activation of enzymes, cell expansion, and the mobilization of stored reserves, which collectively facilitate the transition from dormancy to germination [15]. Gibberellic acid (GA_3_) is a common gibberellin applied as a seed treatment [18,19,20]. It is typically used by imbibing the seeds in a solution containing the hormone through priming [21,22]. However, there are challenges in treating large quantities of seeds through these imbibition methods, as priming can result in a reduction in seed longevity [23,24,25,26,27]. High costs are also incurred with priming through the application itself, drying the seeds after priming, and maintaining the seed in a low-humidity environment for safe storage [26].

Typically, it is more efficient to apply treatments to seeds using seed coating techniques. This approach applies layers of glues and powder materials to the outside of the seed until a desired artificial covering is achieved [9]. Generally, the coating application is applied relatively quickly and does not result in the seed hydrating to a level that will impact seed longevity [9]. The challenge associated with coating seeds with plant growth regulators, like GA_3_, is that the hormone can quickly leach away from the seed. This can be especially true for dormant plantings when the seeds remain in the soil for several months until conditions are suitable for plant growth. Subsequently, GA_3_ may leach from the coating over this period and not produce a treatment response. Additionally, applying high rates of GA_3_ to the seed is typically not advisable because it can result in seedling abnormalities [13].

Polymer-based carriers have found applications in the medical field as vehicles to slowly release active compounds (drugs) into patients over time [28]. In the medical field, drugs are impregnated into a polymer. The drug is released as the polymer breaks down through surface erosion, cleavage of polymer bonds via hydrolysis, or diffusion [28,29]. This approach to drug delivery ensures that a constant amount of the active ingredient remains in the body, reducing the number of doses a patient needs to take [30,31,32,33]. The slow release of the drug through the breakdown of the polymer in the body also leads to improved delivery profiles [29,30]. This type of “slow-release” polymer technology may help keep GA_3_ from leaching away from the seed and deliver the hormone at dose rates that are beneficial to seed germination and effective at breaking dormancy [28]. Outside of the medical field, applications for a slow-delivery polymer system are rare. Polymers used in seed coatings have mainly been used as carriers to apply pesticides, fungicides, fertilizers, and other beneficial ingredients [34,35,36,37]. To our knowledge, there have not been seed coatings applied where the polymer is used to control the release of plant hormones over time.

Our research was conducted using the *Penstemon* (Mitch.) species common to the Great Basin region of the western United States. *Penstemon* is the most species-rich plant genera in the Great Basin and the largest endemic genus in North America [38,39,40]. These species are valued due to their use by pollinators [41,42,43], their visual appearance on the landscape [44], and as early successional species that are capable of colonizing disturbed areas [45,46].

In this work, we first assessed how the performance of different slow-release polymer formulations impregnated with GA_3_ influenced germination and early plant growth on *P. palmeri* A. Gray (Palmer’s penstemon). The different polymers were applied to the seed as a liquid, a powder, or as a combination of the two seed coatings. Upon completion of this study, we then ran the same tests with one of the preferred polymers and application methods on four additional *Penstemon* species, *P. pachyphyllus* A. Gray ex Rydb. (thickleaf penstemon), *P. comarrhenus* A. Gray (dusty penstemon), *P. strictus* Benth. (Rocky Mountain penstemon), and *P. eatonii* A. Gray (firecracker penstemon). We hypothesized that (1) different polymer matrices GA_3_ carriers and the forms in which they were applied to the seed would vary in their ability to overcome seed dormancy and improve early plant growth, and (2) the success of a GA_3_ polymer coating would vary by species and their relative levels of seed dormancy.

## 2. Results

### 2.1. Seed Coating Development on Penstemon palmeri

The form of the polymer used to deliver GA_3_ to the seed had the greatest effect on seed germination (F = 110.4, *P* < 0.001), with an effect size almost three times larger than the type of polymer used (F = 37.0, *P* < 0.001). There were also strong interactions between the polymer form and polymer type (F = 30.7, *P* < 0.001), indicating that the type of polymer used differed in its impact on germination based on the form in which it was applied to the seed. Furthermore, the type of polymer used in the seed coating and its applied form interacted with incubation temperature (Table S1).

**Figure 1:**
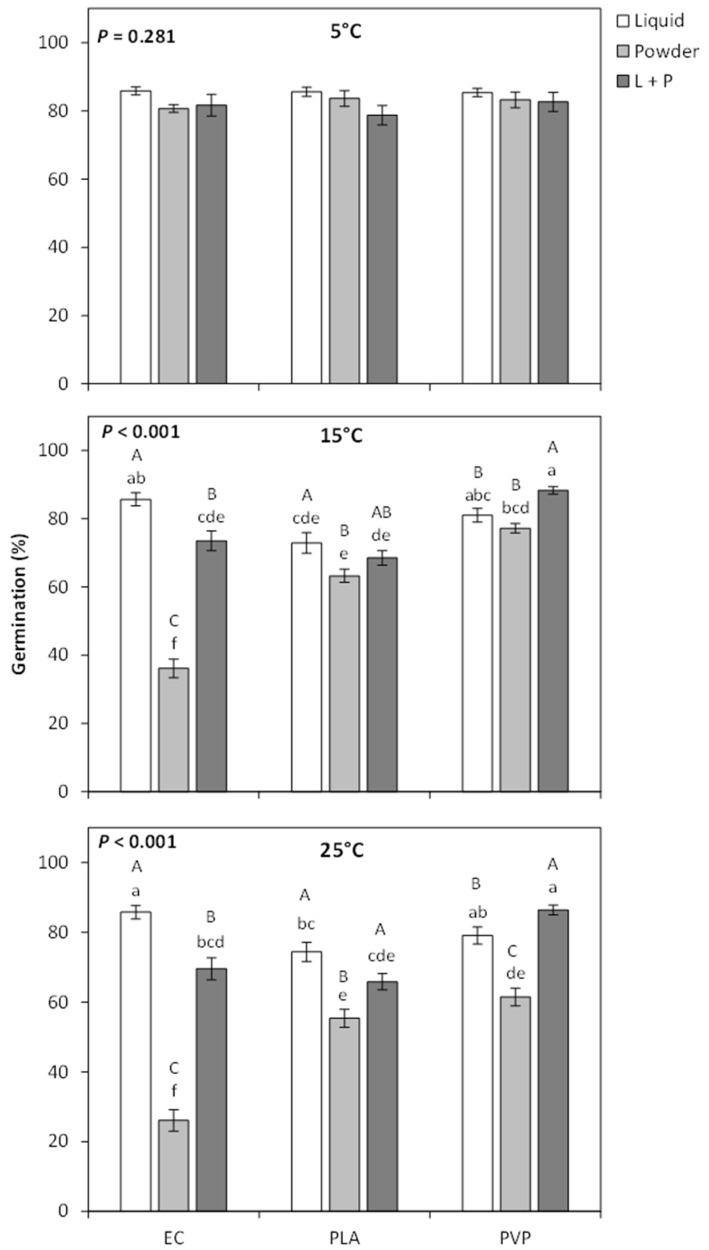
Mean germination percentage (±SE) of *Penstemon palmeri* seed that was treated with either ethylcellulose (EC), polylactic acid (PLA), or polyvinylpyrrolidone (PVP) polymers that were impregnated with gibberellic acid and applied to the seed as a liquid, powder, or a combination of powder and liquid. Seeds were germinated at 5, 10, and 25 °C. Within each temperature graph, differing lowercase letters indicate significant differences across all treatments, and differing capital letters indicate differences between the polymer application forms (*P* < 0.05).

**Figure 2:**
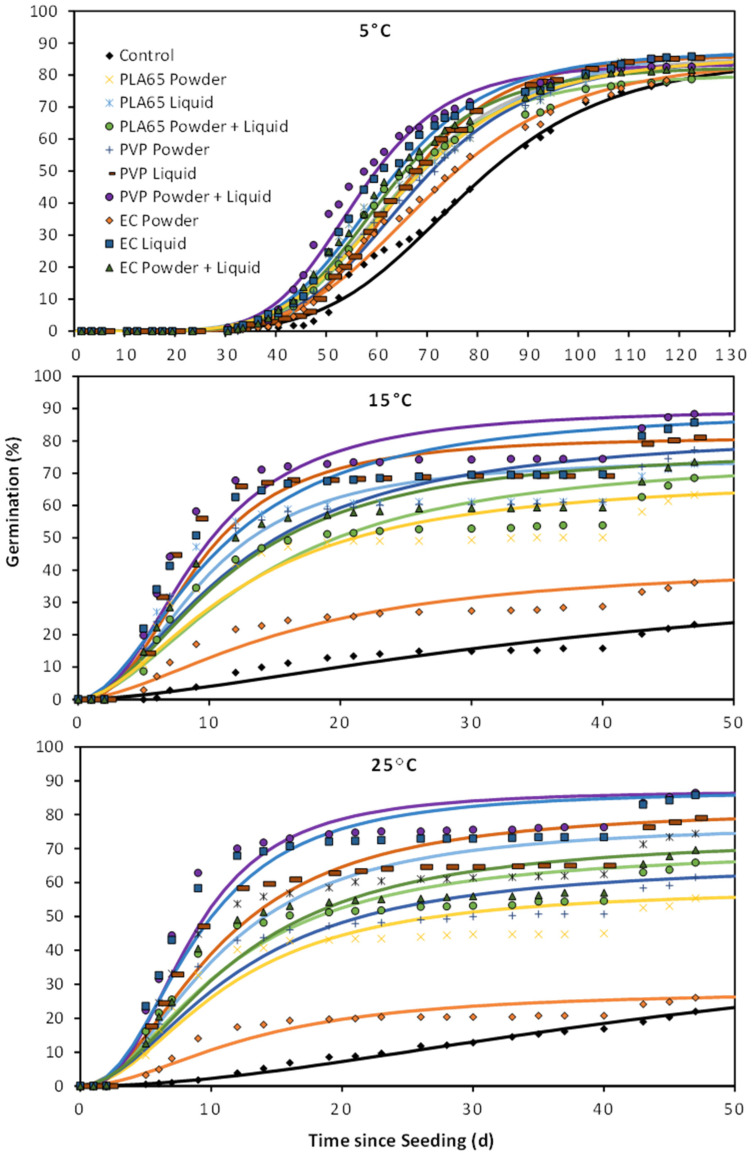
Cumulative germination from seed treatments described in Figure 1 plus seed left untreated (control) and coated with no active ingredient (blank). The solid line represents cumulative germination over time estimated from a three-parameter log–logistic curve, and the symbols indicate germination recorded on a specific day.

At 5 °C, there was no difference in seed germination among the treatments, but strong treatment effects were found from GA_3_ treatments at 15 °C and 25 °C (Figure 1). At 15 °C and 25 °C, applying GA_3_ to the seeds using a liquid coating tended to have higher germination than when GA_3_ was applied as a powder coating (Figure 1). This effect was particularly strong for EC coatings, where the seeds with the liquid coating had a 3.2 times higher rate of germination than the powdered coating. Applying GA_3_ through both a liquid and a powder coating did not improve germination more than just applying a liquid coating for EC and PLA polymers. For PVP coatings, a combination of liquid and polymer treatments had a slight improvement in germination over just applying a liquid treatment (~9% increase in germination at both 15 °C and 25 °C). Overall, the EC liquid coating and PVP with both liquid and powder coating tended to be statistically higher than the other seed treatments (Figure 1). Final seed germination for most GA_3_ treatments was dramatically improved over the control at 15 °C and 25 °C, ranging from 1.6- to 3.0-fold higher germination at 15 °C and 2.5–3.9-fold higher germination at 25 °C. The only treatment not higher than the control was EC powder at 25 °C (Figure 2).

**Figure 3:**
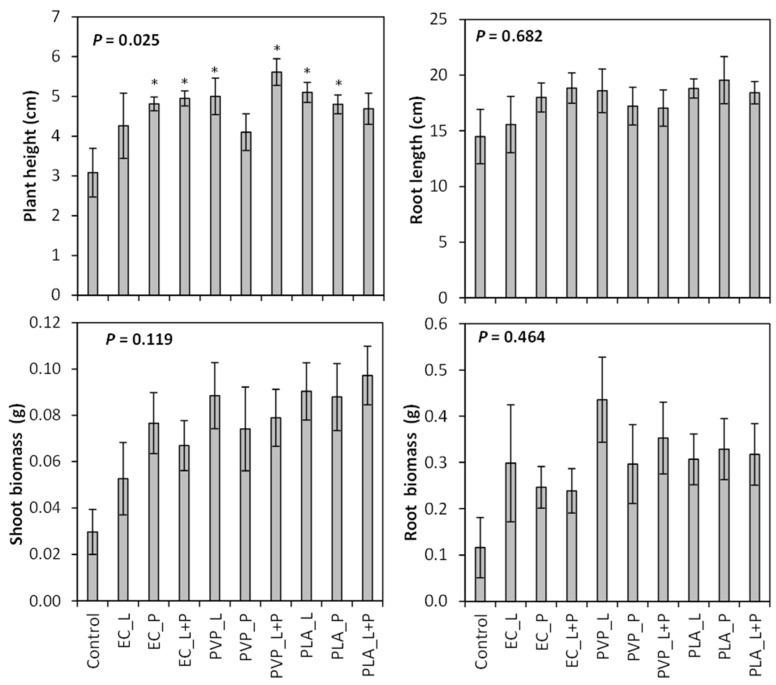
Mean plant height, root length, and root and shoot biomass (±SE) produced from *Penstemon palmeri* seed that was treated with ethylcellulose (EC), polylactic acid (PLA), and polyvinylpyrrolidone (PVP) polymers that were impregnated with gibberellic acid and applied to the seed as a liquid (L), powder (P), or a combination of liquid and powder (L + P). Asterisk indicates difference from the control (*P* < 0.05).

The seed treatments tested in this study resulted in plants with similar root lengths, shoot biomass, and root biomass (*P* > 0.05; Table S2). The shoot biomass results approached significance (*P* = 0.119, Figure 3). When the different seed coatings were compared with the control, plant height was increased for all seed coatings except for the EC liquid coating, PLA liquid + powder coating, and PVP powder coating (Table S3; Figure 3). Those seed coatings that increased shoot height had plants that were 51–82% taller than the control.

### 2.2. Evaluation of a GA_3_ Seed Coating on Additional Penstemon Species

**Figure 4:**
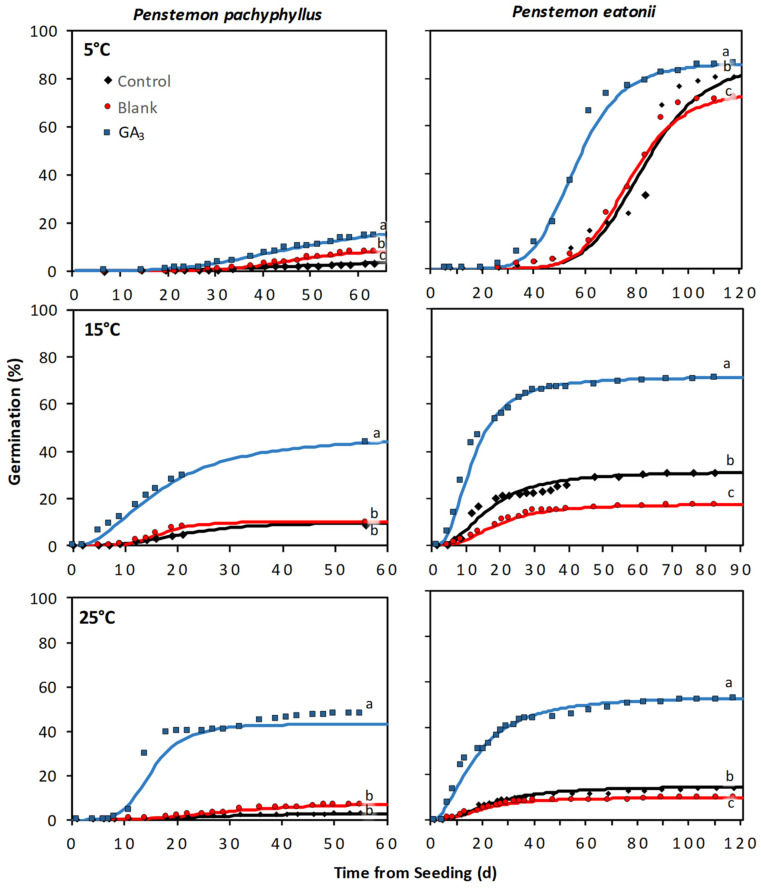
Cumulative germination from *Penstemon pachyphyllus* and *P. eatonii* seeds treated with a liquid EC gibberellic acid (GA_3_) coating, coated with no active ingredient (blank), and left untreated (control). The solid line represents cumulative germination over time estimated from a three-parameter log–logistic curve, and the symbols indicate germination recorded on a specific day. Differing lowercase letters indicate significant differences (*P* < 0.05) among treatments.

**Figure 5:**
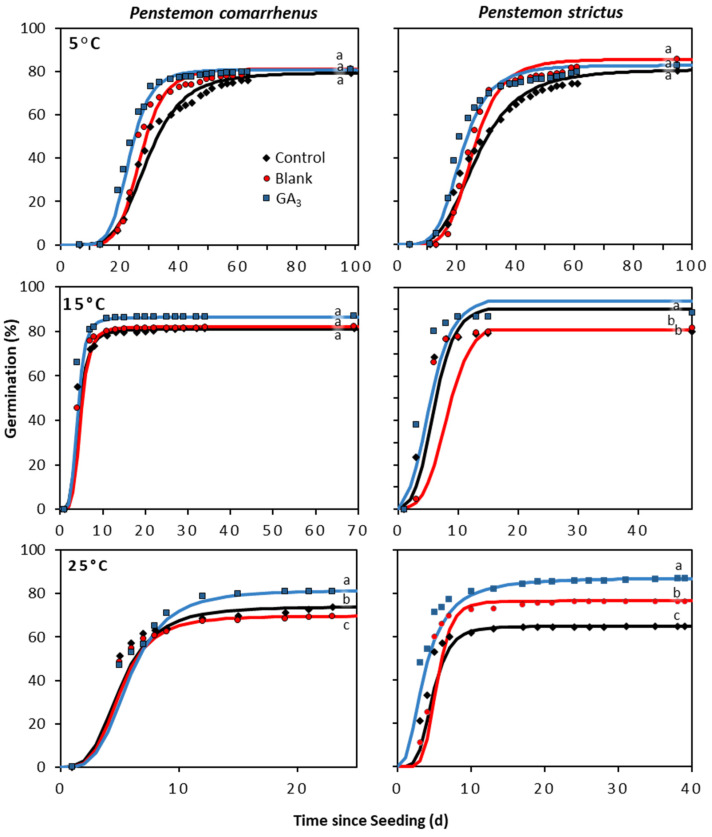
Cumulative germination from *Penstemon comarrhenus* and *P. strictus* seeds treated with a liquid EC gibberellic acid (GA_3_) coating, coated with no active ingredient (blank), and left untreated (control). The solid line represents cumulative germination over time estimated from a three-parameter log–logistic curve, and the symbols indicate germination recorded on a specific day. Differing lowercase letters indicate significant differences (*P* < 0.05) among treatments.

The response of the additional *Penstemon* species to the GA_3_ seed coating varied by species, and for most species, the treatment response was influenced by incubation temperature and the interaction between these two factors (Table S4). The greatest treatment responses between a liquid EC coating and the control tended to be under the warmer incubation temperatures (i.e., 15 and 25 °C) and for those species with high seed dormancy (i.e., *P. pachyphyllus* and *P. eatonii*) (Figure 4 and Figure 5). For *P. pachyphyllus* the GA_3_ coating increased seed germination by 3.3-, 4.0-, and 15.2-fold over the control at 5, 15, and 25 °C, respectively (Figure 4 and Figure 5). Trials on *P. eatonii* also showed that a GA_3_ coating increased germination over the control by 0.1, 1.3, and 2.8-fold at the same respective temperatures. Research on *P. strictus* and *comarrhenus* showed a slight improvement in germination (0.3 and 0.1-fold increase, respectively) over the control at 25 °C. For all species, the blank coating generally responded similarly to the control, but in some instances the blank coating had slightly lower or higher germination than the control, depending on species and incubation temperature (Figure 4 and Figure 5). As with *P. palmeri*, the seed treatments applied to *P. pachyphyllus*, *P. eatonii*, *P. comarrhenus*, and *P. strictus* resulted in plants with similar shoot and root lengths and shoot and root biomass (Table S5). The exceptions were those of *P. eatonii*, where plant shoots were 79% taller than the control, and *P. comarrhenus*, where the blank root weight was 122% more than the control but was similar to the GA_3_ seed coating (Figures S1–S4).

## 3. Discussion

The five *Penstemon* species employed in this study showed various degrees of seed dormancy. As stated previously, seed dormancy can often take multiple growing seasons before it is overcome or delay germination to a period later in the season with unfavorable growing conditions [3,10,11,12]. Effective restoration efforts, however, require rapid seed germination and plant growth to help out-compete invasive species and stabilize soils [47]. This requirement often forces restoration practitioners to pick species with lower seed dormancy [10,11]. Our research has shown that a polymer seed coating impregnated with GA_3_ can alleviate seed dormancy, with the effectiveness of the seed coating influenced by the type of polymer used, the form in which it is applied on the seed, the species used in the evaluation, and the temperatures at which seeds are germinated. All three polymer types tested in this study proved effective at delivering GA_3_ to the seed, as evidenced by improved seed germination. Applying the polymer in a liquid form showed enhanced seed germination over dry powder applications. This enhanced germination may result from an increased contact surface area between the seed and the polymer, which would facilitate a higher and more consistent rate of delivery of GA_3_ to the seed. Additionally, the treatment response may be due to the liquid coating penetrating the seed at the time of coating, whereas the dry powder is only attached to the outside of the seed. However, increasing GA_3_ concentrations onto the seed is the least plausible answer because when the polymer was applied as both a liquid and a powder, we did not see any significant additive effects due to the concentration of GA_3_ being doubled for this treatment.

The liquid, EC, GA_3_ seed coating was among the top treatments for seed germination response among the several different seed coating formulations tested in this research trial. This liquid, EC, GA_3_ seed coating formulation may be particularly valuable in systems where seeds are sown and remain in the soil for a prolonged period because of the EC polymer’s ability to prolong the release of active ingredients. Based on the results of our study, research is now merited to test a liquid, EC, GA_3_ seed coating, and potentially other polymer coatings under field conditions.

In our evaluation of different GA_3_ seed coatings, there was a minimal treatment response relative to untreated seeds at 5 °C, but there was a strong response at 15 °C and 25 °C. In our study, at 5 °C, minimal germination occurred until approximately 7 weeks, and then germination was rapid. Presumably, it was at this time that the cold stratification period had overcome seed dormancy, which allowed for high seed germination and, subsequently, a minimal response to a GA_3_ seed treatment. This response is similar to a stratification study by Alan and Meyer (1990) [48], where they showed an increase in germination of three *Penstemon* species following an 8-week stratification. In our research trial, *P. palmeri*, *P. pachyphyllus*, and *P. eatonii* had higher levels of dormancy than *P. comarrhenus* and *P. strictus*. As predicted by our hypothesis, these species with higher seed dormancy levels had the greatest benefit from the polymer GA_3_ seed coatings, with a 1.3–15.2-fold increase in germination when studies were conducted at 15 or 25 °C. The species with low dormancy tended to have high germination across all treatments. Thus, the results of our study seem to indicate that the polymer GA_3_ coating will have the greatest effect on species with high dormancy.

The polymer GA_3_ coating developed from our research may also be most effective in sites or planting years that do not experience prolonged moist, cool periods to break seed dormancy. In our study at lower temperatures (5 °C), we generally observed minimal to no change in the germination efficiency between treated and untreated seeds. This temperature effect may be partly due to the seeds stratifying under this cold temperature. It is common for many plant species from colder climates to need a period of cold stratification to break dormancy and germinate [49,50]. Restoration seedings can fail in warm, dry years when environmental conditions are inadequate to overcome seed dormancy [11,51,52]. Climate change may further exacerbate restoration failures in the future, as it is expected to warm soils during the winter dormancy break period [53]. Subsequently, a polymer GA_3_ seed coating may potentially improve restoration efforts in sites or planting years that do not experience prolonged moist, cool periods to break seed dormancy. This treatment may become even more critical with warming soil conditions in the winter period.

While our polymer GA_3_ coating enhanced germination by breaking seed dormancy, we worried that treating the seed with high rates of GA_3_ would cause abnormal plant growth. GA_3_ is known to cause damage to the root system by stunting roots and can lead to the swirling of stems and excessive shoot growth [13]. Our growth studies show that our treatments do not negatively impact the plant’s growth. Treated seeds generally did not have significantly different growth profiles (i.e., root and shoot length and below- and above-ground biomass) than the control or blank coated seeds. These findings allow us to conclude that this polymer GA_3_ technology can be used to improve the germination of species that are difficult to germinate while not interfering with the natural processes of plant growth beyond germination.

## 4. Materials and Methods

### 4.1. Study Species 

The seed used in the study was supplied by the Utah Division of Wildlife Resources, Great Basin Research Center and Seed Warehouse (Ephraim, UH, USA), and had a viability ranging from 86 to 97% and purity of 84–98% (Table S6). Of the five *Penstemon* species used in the study, *P. palmeri* has the widest range, with plants extending from Oregon down through Idaho to New Mexico and from California to Colorado. *Penstemon comarrhenus* has the smallest range, primarily occurring in Nevada through the Four Corners region. Each of these *Penstemon* species provides unique ecological functions, such as their ability to serve different types of pollinators. *Penstemon palmeri* flowers are white to lavender-pink with stark red-violet lines on the lower lip reaching into the throat of the flower. This species commonly attracts bumblebees (*Bombus* sp.), carpenter bees (*Xylocopa* sp.), and digger bees (*Anthophorini tribe*) [42]. *Penstemon pachyphyllus* flowers are large and purple and are often pollinated by large bumblebees (*Bombus* sp.) [41]. *Penstemon comarrhenus* has pale blue flowers with light pink throats and possesses traits that will attract a variety of bee syndromes [42,43,49,54]. *Penstemon strictus* flowers are deep blue to purple with a white tone towards the flower’s throat. The flowers also include red-violet lines running down the throat of the flower. This species is an important nectar source for various bumblebees [55]. The species is also visited by other bees and wasps and occasionally by hummingbirds [53]. *Penstemon eatonii* flowers are bright red and have a narrow corolla tube compared to the other *Penstemons*. This flower is highly sought after by hummingbirds [42,49,54].

### 4.2. Seed Coating Development on Penstemon palmeri

Commercially available polymers that allow for the slow release of an active ingredient are varied and extensive. We evaluated polymers that were biodegradable and biocompatible to limit the amount of microplastics released into the environment. Polymers tested included polylactic acid (PLA) at a 65 molecular weight (Poly L Lactic Acid Biopolymer™, Goodfellow, Huntingdon, UK), ethylcellulose (EC) (Ethocel™, Dow Chemical, Midland, MI, USA), and polyvinylpyrrolidone (PVP) (Agrimer 15™ Ashland Inc., Covington, KY, USA). These polymers were impregnated with GA_3_ by dissolving the polymer in a solvent, adding GA_3_, and then removing the solvent. The impregnated polymers were then applied to the seed as a solution of the dissolved polymer/GA_3_ (liquid), as a powder after solvent removal, or as a combination of the two treatments (powder and liquid).

Polylactic acid is a thermoplastic polymer that will degrade into lactic acid. The addition of lactic acid into soil has been shown to improve soil fertility parameters by increasing *Lactobacillus* activity [54,56]. Ethylcellulose is a linear polysaccharide chain composed of cellulose derivatives, where hydroxy (−OH) function groups have been replaced with ethoxy groups (−OCH_2_CH_3_). This polymer has hydrophobic intermolecular interactions that prolong the release of active ingredients. Ethylcellulose polymers are commonly used in the food industry for packaging and in specialty coatings in the pharmaceutical industry [57]. Polyvinylpyrrolidone is synthesized from its monomer, *N*-vinylpyrrolidone. This polymer is used in various food, hygiene, and medical applications, often as an additional filler [58].

The polymer coating formulation was designed to treat 100 g of seed. To impregnate the polymers with GA_3_, we first dissolved 4.68 g of the polymer material in 50 mL of dichloromethane (PLA, EC, and PVP). In a separate vial, 0.83 g of GA_3_ was added to ethanol. The polymer and GA_3_ solutions were then combined and mixed on a stir plate until a homogenous mixture was achieved. The final polymer solutions were slightly opaque and light yellow in color. These polymer solutions could be used directly as a binder when performing seed coatings, or the impregnated polymer could be precipitated from solution and added to the seed coatings in powder form.

Powdered GA_3_ polymer was prepared by taking the final polymer solution obtained above and adding it to hexanes (dropwise) to precipitate the polymer. The precipitate was then filtered, air dried, finely ground using an Oster kitchen blender, and then passed through a flour mill to produce a fine powder.

Seeds were coated in 100 g batches with different treatments in a 31-cm diameter rotary-drum seed coater (Universal Coating Systems, Independence, OR, USA). For the liquid polymer treatment, we first added 50 mL of the GA_3_ polymer solution to the seeds and then let the seeds spin in the seed coater until dry (~180 s). We then added a second coating layer comprised of 60 mL of PVP in water made with a 40% solid content and 175 g of calcium carbonate powder (Clayton Calcium, Parma, ID, USA). Seeds coated in this fashion contained ~57 mg polymer and 8.3 md GA_3_ per gram of seed.

Seeds treated with the powdered GA_3_-impregnated polymer received the same amount of PVP binder (60 mL of a 40% solution in water) and calcium carbonate powder as used with the liquid GA_3_ polymer treatment, and the GA_3_-impregnated polymer powder (~5 g as prepared above) was mixed in with the calcium carbonate powder. Treatments receiving both liquid and powder GA_3_ polymers had the liquid GA_3_ polymer applied directly to the seed and the powdered GA_3_ polymer mixed in with the calcium carbonate powder and added after the addition of the PVP solution. With the combined liquid and powder treatment, the seed received twice the GA_3_ as when only the liquid or the powder GA_3_ polymer treatment was applied.

In addition to the different GA_3_ polymer treatments described above, we also had a treatment where the seeds were coated with the same amount of PVP and calcium carbonate powder, but no GA_3_ polymer was applied (identified as “blank”), which served as a procedural control. Additionally, untreated seeds were used as a control. This study design gave us a total of 11 seed treatments in the study (3 polymer chemistries × 3 polymer application methods plus a control, and blank treatments). All seed coatings evaluated in the study were placed on a forced air dryer (Universal Coating Systems, Independence, OR, USA) at 36 °C for 15 min.

We assessed the impact of the individual seed treatments on germination in the laboratory at three different temperatures (5, 15, and 25 °C). For each temperature, there were 8 replicate Petri dishes per treatment for a total of 264 Petri dishes used in the study (3 temperatures × 11 seed treatments × 8 replications).

Petri dishes were 15 cm in diameter and contained 35 seeds with two layers of moistened blotter paper. Petri dishes were placed into Ziplock bags to maintain moisture and placed in Precision Plant Growth Chambers (Thermal Fischer Scientific, Waltham, MA, USA) with 12 h light/dark intervals. Seed germination was generally counted every 2–3 days. Petri dishes were re-randomized within the growth chamber each time germination was assessed. Germination trials were run until no germination was detected for most of the treatments for one week.

Plant growth was assessed for the same seed treatments tested in the germination study in a randomized block design composed of 10 replicate blocks. Plants were grown in a 3:1 mix by volume of soil and vermiculite (Therm-O-Rock West, Inc., Chandler, AZ, USA) in 38 mm diameter × 210 mm deep cone-tainer pots. Soil was collected near Santaquin Utah (lat 39°54′35″ N, long 111°48′45″ W). Here, the soil is classified as a Donnardo stony loam (42% sand, 38% silt, and 20% clay), with a pH of 7.4–7.8 and 1–3% organic matter [59].

Each pot contained 10 seeds that were gently pressed so the top of the seed was level with the soil surface. Pots were incubated in a walk-in growth chamber (Environmental Growth Chambers, Chagrin Falls, OH, USA), held at a constant temperature of 25 °C with a humidity of approximately 65%. Lights in the chamber provided a 12 h photoperiod, with a maximum photosynthetically active radiation flux density of approximately 780 µmol m^−2^s^−1^, at plant height. Soil in the pots were kept moist by watering approximately 3 times/week to field capacity. Prior to each watering, three random pots were weighed, and the average amount of water needed to bring the pots to field capacity was applied to all of the pots. Seedling emergence was counted each week, and the pots were re-randomized within each block. After four weeks, soil was washed from the roots, and root and shoot length were measured. The above- and below-ground biomass of each plant was also harvested (oven dried for 72 h at 65 °C).

### 4.3. Evaluation of a GA_3_ Seed Coating on Additional Penstemon Species

We evaluated the influence of a GA_3_ seed coating that was made with an EC polymer and applied to the seed as a liquid on *P. pachyphyllus*, *P. comarrhenus*, *P. strictus* and *P. eatonii* seeds. This formulation was chosen because it was one of the top-performing treatments in the previous study, and we anticipate that an EC polymer would provide a more extended release of GA_3_ relative to the other polymers in the study due to its relatively strong hydrophobic nature. Methods for applying this coating were the same as described previously. Separate germination and plant biomass trials were performed following the same procedures that were used for *P. palmeri.* In each trial, the GA_3_ seed coating was compared to a blank coating and untreated control seeds.

### 4.4. Statistical Analysis

For the seed coating development trial on *P. palmeri*, we assessed the influence of the different polymer coatings on seed germination and above- and below-ground biomass using generalized linear mixed-effects models (GLM) in JMP Pro (version 16 SAS Institute, Inc., Cary, NC, USA). In the models, polymer type, the form in which the polymer was applied, incubation temperature, and their interactions were included as fixed effects, and block was a random effect. For germination data, the models were fitted with a binomial response distribution with a Logit link, and the biomass data were fitted with a Poisson response distribution with a log link [60]. The blank and control treatments were not included in these analyses. Pairwise comparisons were performed to assess differences among the treatments within an incubation temperature using the Tukey–Kramer honestly significant difference multiple comparison method. When appropriate, pairwise comparisons were also performed within each incubation temperature and polymer type to compare differences between the different forms in which the GA_3_ polymer was applied to the seed.

We also used a GLM to assess differences among all seed treatments, including the blank and control treatment. Again, block was considered a random effect, and pairwise comparisons were performed to assess differences between treatments with models fit with the appropriate distribution as previously described. This same procedure was also used for the second part of the study for the analysis of the GA_3_ seed coating on multiple *Penstemon* species. Here, separate analyses were made on the control, blank, and GA_3_ seed coating treatment for each species evaluated in the study.

## 5. Patents

Larson, A.; Michaelis, D.J.; Madsen, M.D., 2022. Development and Use of a Slow-Release Polymer Seed Coating System to Deliver Growth Hormones for Enhancing Seed Germination and Early Plant Growth. Provisional Patent Application No. 63317605.

## Data Availability

The data presented in this study are openly available at Brigham Young University ScholarsArchive at https://scholarsarchive.byu.edu/data/57/ (accessed on 3 October 2023).

## Supplementary Materials

The following supporting information can be downloaded at https://www.mdpi.com/article/10.3390/plants12244139/s1, Table S1. *F* and *P* values show the impact on *Penstemon palmeri* seed germination from the type of polymer used to deliver gibberellic acid, the form the polymer is applied, the temperature seeds are incubated, and the interactions of these effects. Table S2. *F* and *P* values show the impact on *Penstemon palmeri* shoot height, root length, shoot biomass, and root biomass, from seed treated with different types of polymers containing gibberellic acid, with polymers applied to the seed in different forms and the interactions of these effects. Table S3. *F* and *P* values show the impact of the different seed treatments evaluated in the study (including untreated seed) on *Penstemon palmeri* final germination percentage (FGP) at 5, 15, and 25 °C and time to 50% germination at 5 °C. The table also shows the impact of the treatments on shoot height, root length, shoot biomass and root biomass. Table S4. *F* and *P* values show the influence of seed treatment and incubation temperature, and their interaction on seed germination of *Penstemon pachyphyllus, P. eatonii, P. comarrhenus,* and *P. strictus*. Table S5. *F* and *P* values show the influence of seed treatment on plant height, root length, and root and shoot biomass for *Penstemon pachyphyllus, P. strictus, P. comarrhenus,* and *P. eatonii*. Figure S1. Mean plant height, root length, and root and shoot biomass (±SE) produced from *Penstemon pachyphyllus* seed that was either treated with an ethylcellulose gibberellic acid (GA_3_) seed coating, coated with ethylcellulose but no GA_3_ (blank), and left untreated (control). Differing lowercase letters indicate significant differences (*P* < 0.05) among treatments. Figure S2. Mean plant height, root length, and root and shoot biomass (±SE) produced from *Penstemon comarrhenus* seed that was either treated with an ethylcellulose gibberellic acid (GA_3_) seed coating, coated with ethylcellulose but no GA_3_ (blank), and left untreated (control). Differing lowercase letters indicate significant differences (*P* < 0.05) among treatments. Table S6. Penstemon species used in the research trial and if applicable the species variety or location where seeds were collected and the seed test viability and purity. Seed was obtained from the Utah Division of Wildlife Resources, Great Basin Seed Warehouse.
