# Supplementary material for: Slow Release of GA3 Hormone from Polymer Coating Overcomes Seed Dormancy and Improves Germination
Source: Plants (Basel). 2023 Dec 12;12(24):4139. doi: 10.3390/plants12244139 (PMC10748187; doi:10.3390/plants12244139)
Supplement: Supplementary file 1 [file plants-12-04139-s001.zip › plants-2699804-supplementary.pdf]

Supplementary Material

Table S1. *F* and P values show the impact on *Penstemon palmeri* seed germination from the type of polymer used to deliver gibberellic acid, the form the polymer is applied, the temperature seeds are incubated, and the interactions of these effects.

Effect	*F*	P
Polymer Type (P)	37.0	< 0.001
Polymer Form (F)	110.4	< 0.001
Temperature (T)	75.4	< 0.001
P X F	30.7	< 0.001
P X T	7.1	< 0.001
F X T	23.8	< 0.001
P X F X T	4.6	< 0.001

Table S2. *F* and P values show the impact on *Penstemon palmeri* shoot height, root length, shoot biomass, and root biomass, from seed treated with different types of polymers containing gibberellic acid, with polymers applied to the seed in different forms and the interactions of these effects.

Effect	*F*	P
	Shoot Height	
Polymer Type (P)	0.3	0.739
Polymer Form (F)	1.3	0.285
P X F	1.8	0.149
	Root Length	
Polymer Type (P)	0.8	0.432
Polymer Form (F)	0.1	0.873
P X F	0.9	0.487
	Shoot Weight	
Polymer Type (P)	2.7	0.072
Polymer Form (F)	0.1	0.895
P X F	0.6	0.672
	Root Weight	
Polymer Type (P)	0.5	0.591
Polymer Form (F)	1.3	0.283
P X F	1.0	0.434

Table S3. *F* and P values show the impact of the different seed treatments evaluated in the study (including untreated seed) on *Penstemon palmeri* final germination percentage (FGP) at 5, 15, and 25°C and time to 50% germination at 5°C. The table also shows the impact of the treatments on shoot height, root length, shoot biomass and root biomass.

Effect	*F*	P
FGP @ 5°C	1.4	0.205
FGP @ 15°C	64.4	< 0.001
FGP @ 25°C	58.7	< 0.001
Shoot height	2.2	0.025
Root length	0.8	0.613
Shoot biomass	1.6	0.128
Root biomass	1.1	0.381

Table S4. *F* and P values show the influence of seed treatment and incubation temperature, and their interaction on seed germination of *Penstemon pachyphyllus, P. eatonii, P. comarrhenus,* and *P. strictus*.

Species	Effect	*F*	P
P. pachyphyllus	Treatment (Tr)	141.0	< 0.001
	Temperature (T)	10.1	< 0.001
	Tr X T	2.3	0.069
P. eatonii	Treatment (Tr)	175.0	< 0.001
	Temperature (T)	324.7	< 0.001
	Tr X T	14.8	< 0.001
P. strictus	Treatment (Tr)	9.0	< 0.001
	Temperature (T)	21.7	< 0.001
	Tr X T	6.2	< 0.001
P. comarrhenus	Treatment (Tr)	5.6	0.005
	Temperature (T)	1.6	0.209
	Tr X T	12.0	< 0.001

Table S5. *F* and P values show the influence of seed treatment on plant height, root length, and root and shoot biomass for *Penstemon pachyphyllus, P. strictus, P. comarrhenus,* and *P. eatonii*.

	P. pachyphyllus		*P. strictus*		*P. comarrhenus*		*P. eatonii*	
Effect	*F*	P	*F*	P	*F*	P	*F*	P
Shoot length	1.2	0.335	0.1	0.935	1.6	0.222	30.3	< 0.001
Root length	1.5	0.269	1.7	0.206	0.5	0.624	1.0	0.390
Shoot weight	0.3	0.743	0.7	0.514	0.8	0.453	1.8	0.186
Root weight	0.3	0.714	1.7	0.207	4.0	0.031	0.9	0.439
Root/shoot ratio	0.2	0.822	1.7	0.207	2.7	0.085	0.2	0.840

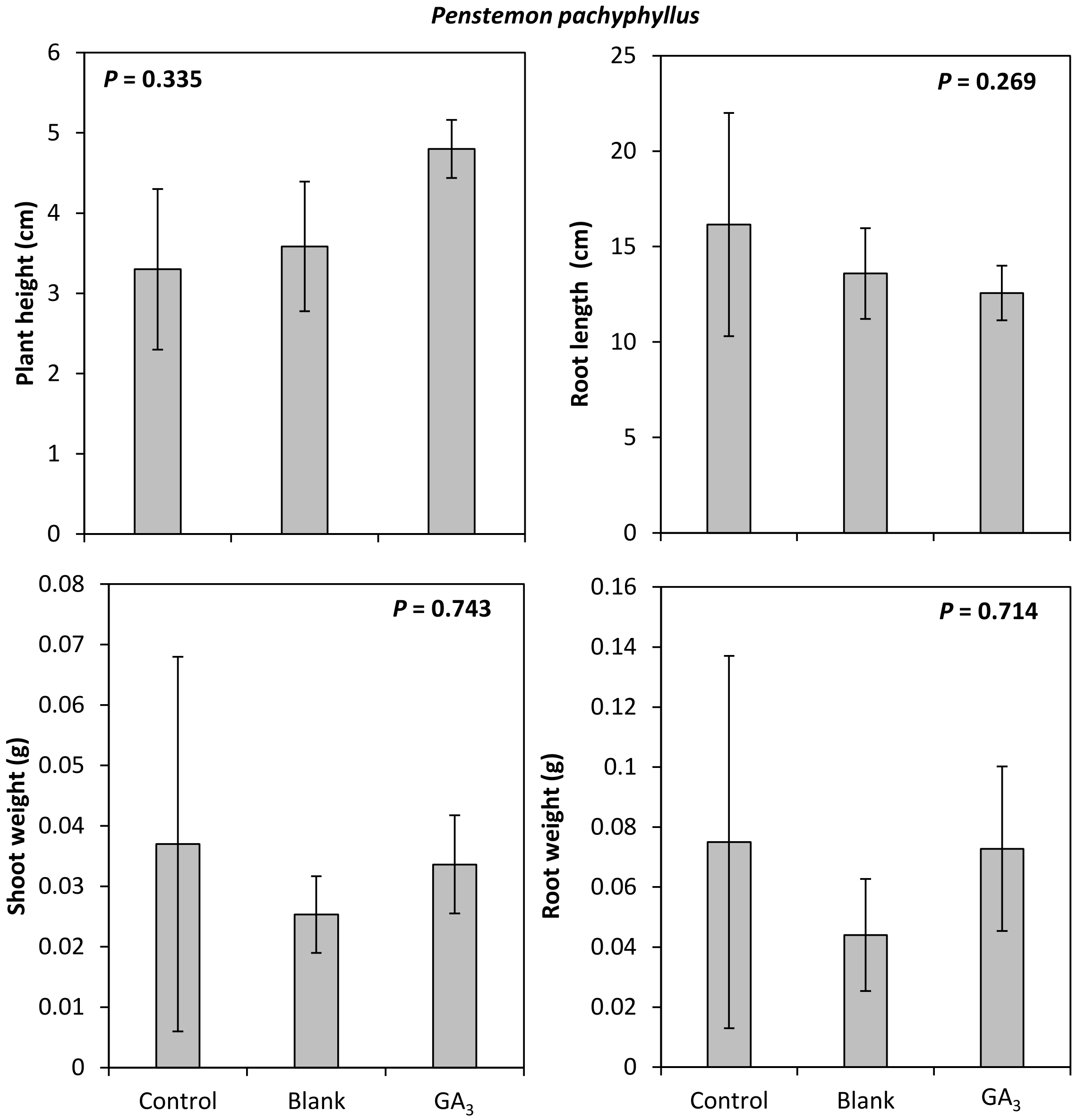

Figure S1. Mean plant height, root length, and root and shoot biomass (±SE) produced from *Penstemon pachyphyllus* seed that was either treated with an ethylcellulose gibberellic acid (GA_3) seed coating, coated with ethylcellulose but no GA_3 (blank), and left untreated (control). Differing lowercase letters indicate significant differences ($P < 0.05$) among treatments.

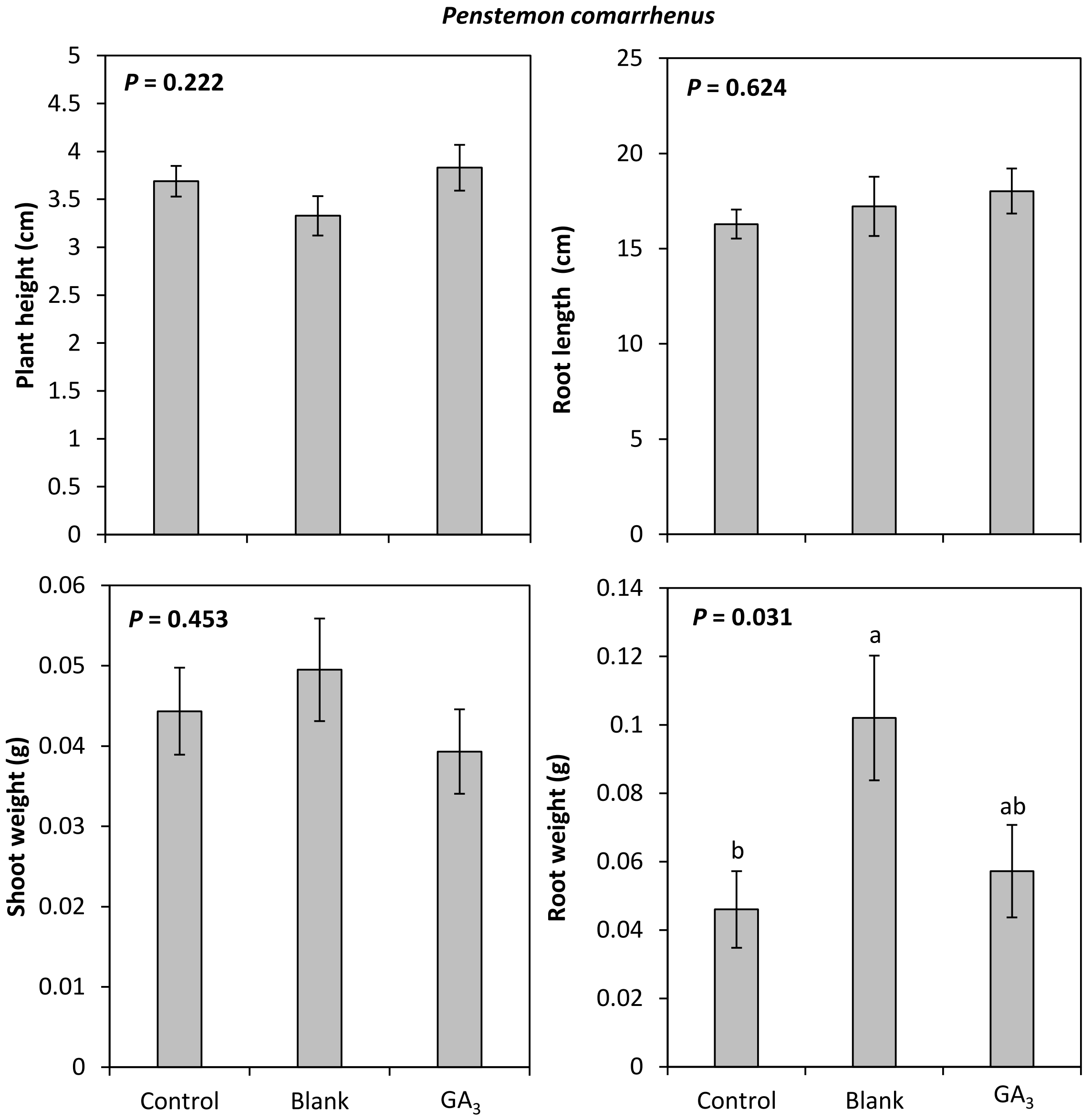

Figure S2. Mean plant height, root length, and root and shoot biomass (±SE) produced from *Penstemon comarrhenus* seed that was either treated with an ethylcellulose gibberellic acid (GA_3) seed coating, coated with ethylcellulose but no GA_3 (blank), and left untreated (control). Differing lowercase letters indicate significant differences ($P < 0.05$) among treatments.

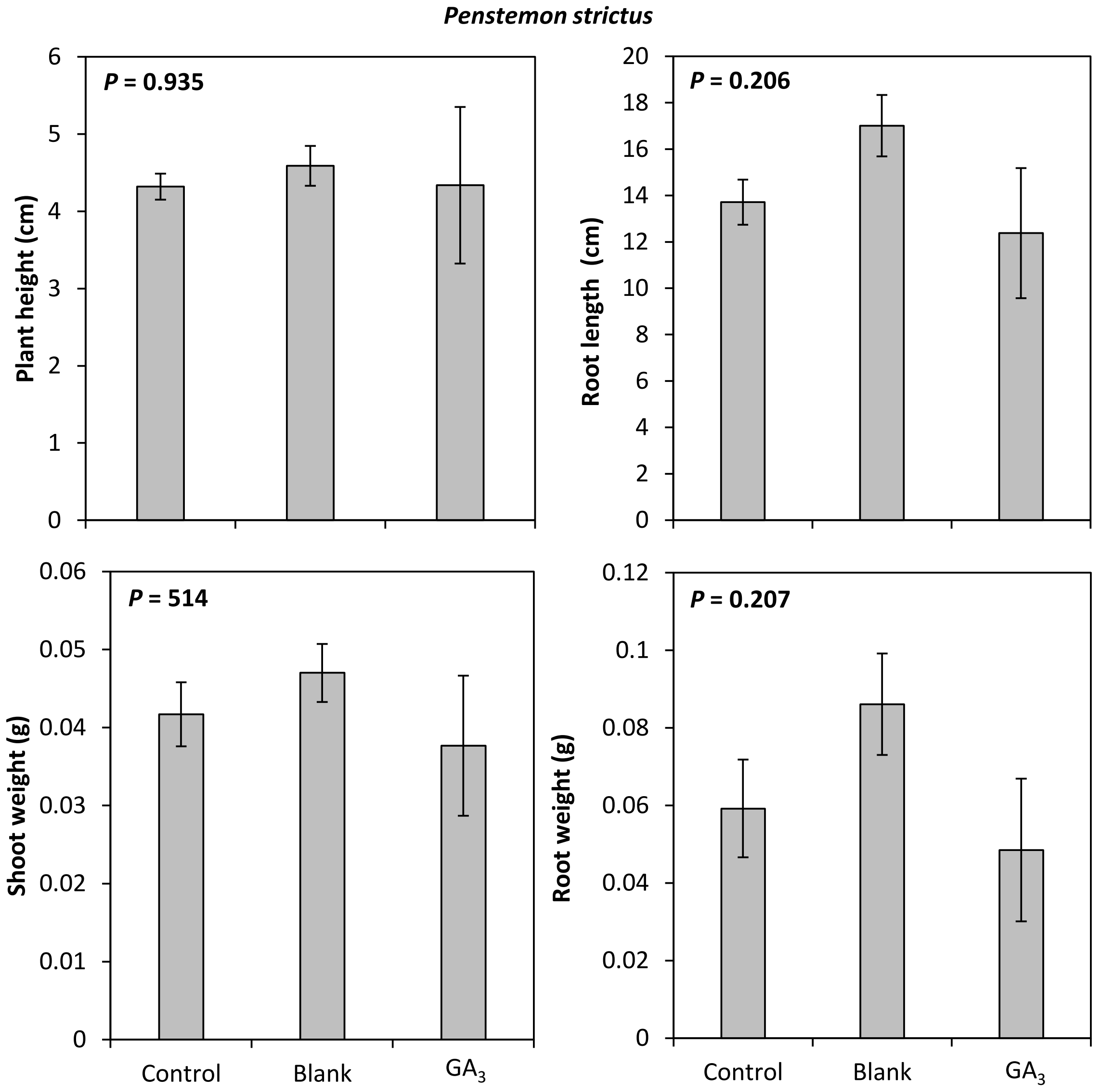

Figure S3. Mean plant height, root length, and root and shoot biomass (±SE) produced from *Penstemon strictus* seed that was either treated with an ethylcellulose gibberellic acid (GA_3) seed coating, coated with ethylcellulose but no GA_3 (blank), and left untreated (control). Differing lowercase letters indicate significant differences (P < 0.05) among treatments.

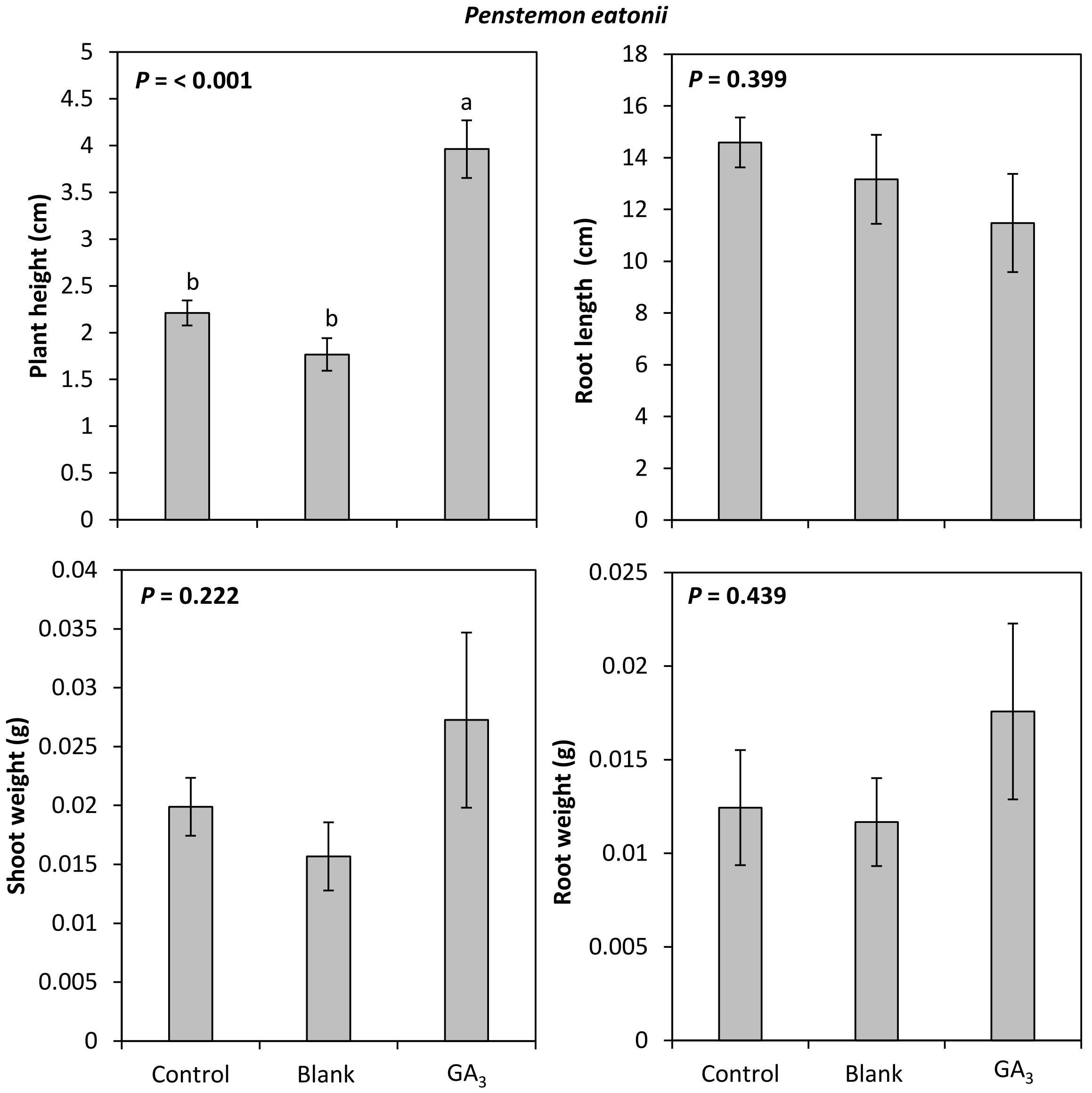

Figure S4. Mean plant height, root length, and root and shoot biomass (±SE) produced from *Penstemon eatonii* seed that was either treated with an ethylcellulose gibberellic acid (GA_3) seed coating, coated with ethylcellulose but no GA_3 (blank), and left untreated (control). Differing lowercase letters indicate significant differences (P < 0.05) among treatments.

Table S6. *Penstemon* species used in the research trial and if applicable the species variety or location where seeds were collected and the seed test viability and purity. Seed was obtained from the Utah Division of Wildlife Resources, Great Basin Seed Warehouse.

Species	Variety	County (State) of Origin	Viability	Purity
palmeri		Beaver (Utah)	97	97.05
pachyphyllus		White Pine (Nevada)	91	92.71
comarrhenus		Kane (Utah)	88	95.53
strictus	'Bandera'		86	97.77
eatonii	'Richfield'		94	83.78
